# VEEG monitoring and electrographic seizures in 232 pediatric patients in ICU at a tertiary hospital in China

**DOI:** 10.3389/fneur.2022.957465

**Published:** 2022-11-23

**Authors:** Tian Sang, Ying Wang, Ye Wu, Qiao Guan, ZhiXian Yang

**Affiliations:** Pediatric Department, Peking University First Hospital, Beijing, China

**Keywords:** electroencephalography, electrographic seizures, status epilepticus, PICU, EEG background

## Abstract

**Objectives:**

To investigate neonatal electroencephalography (EEG) background activity and electrographic seizures in patients in the pediatric intensive care unit (PICU) who underwent bedside video-electroencephalography (vEEG) monitoring.

**Methods:**

A total of 232 pediatric patients admitted or transferred to PICU that underwent vEEG monitoring were retrospectively enrolled in this study, and electrographic status epilepticus was observed after vEEG monitoring.

**Results:**

The median age was 1.56 years [95% confidence interval (CI) = 1.12–2.44]. Electrographic seizures occurred in 88 patients (37.9%), out of which 36 cases (40.9%) had electrographic status epilepticus. Prior epileptic encephalopathy diagnosis [odds ratio (OR) = 6.57, 95% CI = 1.91–22.59, *p* = 0.003], interictal epileptiform discharges (OR = 46.82, 95%CI = 5.31–412.86, *p* = 0.0005), slow disorganized EEG background (OR = 11.92, 95%CI = 1.31–108.71, *p* = 0.028), and burst-suppression EEG background (OR = 23.64, 95%CI = 1.71–327.57, *p* = 0.018) were the risk factors for electrographic seizures' occurrence. Of the 232 patients, the condition of 179 (77.2%) patients improved and they were discharged, 34 cases (14.7%) were withdrawn, and 18 cases (7.8%) died. The in-hospital death rate was 47.6% (10 in 21 cases) in patients with attenuated/featureless, compared to 0/23 with normal EEG background.

**Conclusions:**

Electrographic status epilepticus occurs in more than one-third of patients with electrographic seizures. vEEG is an efficient method to determine electrographic seizures in children. Abnormal EEG background activity is associated with both electrographic seizures' occurrence and unfavorable in-hospital outcomes.

## Introduction

In critically ill patients, epileptic seizures, with the majority being non-convulsive seizures (NCS), may be difficult to detect due to the subtle or the absence of clinical signs (1–5). They can be caused by unhealed status epilepticus (subclinical seizures), acute neurologic impairment (encephalitis, trauma, stroke, and anoxia), and systemic disorders (metabolic disorders, cardiorespiratory disease, poisoning, and sepsis).

Video-electroencephalography (vEEG) monitoring is increasingly being utilized in the identification of epileptic seizures in clinical practice, especially in patients with impaired consciousness (6–9). Electrographic seizures (seizure on EEG) have an estimated occurrence of 20–40% among children who underwent vEEG monitoring in pediatric intensive care unit (PICU) (2, 3, 7, 8, 10). Kirkham et al. (11) reported that either clinical or subclinical seizures are associated with poor survival outcomes in patients in PICU. Meanwhile, studies indicated that the occurrence of status epilepticus in PICU is associated with higher in-hospital mortality (7, 8, 12). EEG has been reported to affect clinical management in about 59% of monitored children, most often by affecting anticonvulsant utilization (13). All these findings inform us that vEEG monitoring in PICU plays an important role in both detecting subclinical seizures and guiding the treatment with anticonvulsants in critically ill patients.

The aim of the present study was to describe current pediatric critical care vEEG utilization using a single-center retrospective study of consecutive patients. According to the continuous vEEG monitoring in PICU, we retrospectively investigated the prevalence of seizures, determined the risk factors for seizures, and further analyzed the association between seizures and clinical outcomes.

## Methods

### Study population

From July 2013 to June 2018, this retrospective study was conducted on all consecutive patients who underwent vEEG monitoring in PICU of Peking University First Hospital, China. This study was approved by the Ethics Committee of the Peking University First Hospital, China (No. 2021KY422). If the patient received multiple vEEG monitorings, the vEEG recording with the poorest performance was registered. Patients with a vEEG monitoring of < 4 h were excluded. Premature infants with a gestational age < 44 weeks and term infants < 28 days after birth were excluded.

### Demographic and clinical information

Demographic and clinical information was gathered from the patients, including age, gender, hospitalized time in PICU, prior diagnosis of epileptic encephalopathy, prior diagnosis of epilepsy, clinical seizures prior to vEEG monitoring, mental status before vEEG monitoring, prior developmental delay or intellectual disability, and acute or systemic disorders. Acute or systemic disorders include neurologic disorder, central nervous system (CNS) infection, autoimmune diseases, sepsis, previous hypoxic ischemic encephalopathy (HIE), other structural brain impairments, mitochondrial encephalomyopathy (ME), and inborn errors of metabolism (IEM).

### vEEG analysis

Bedside vEEG monitoring was performed on each patient using a Nihon Kohden digital video-EEG 1200C instrument (Nihon Kohden, Japan), which consists of an EEG-1200c system program, an isolated power supply unit, an electric J input box, a mini extension junction box, an input converter, a signal switching box, an additional electrical stimulation unit, a light stimulation control unit, a flash lamp, a camera image input unit, a wire assembly, a peripheral capillary oxygen saturation (SpO_2_) adapter, a reusable pulse oxygen probe, a CO_2_ sensor, a nose adapter, and digital video software. The sensitivity is 10–1,500 μV/cm. The EEG electrodes were positioned over the scalp according to the International 10–20 System. All individuals also underwent polyelectromyography (PEMG) during the vEEG monitoring. The occurrence and characteristics of seizures, interictal epileptiform discharges, and EEG background were collected from the vEEG recording. Electrographic seizures were recognized according to the description given in a previous literature (14). Electrographic status epilepticus refers to single or recurrent electrographic seizures that last longer than 30 min in any 1 h epoch. Interictal epileptiform discharges refer to sharp/sharp–slow waves, spikes/ spike–slow waves, polyspike slow waves, and paroxysmal fast activity. EEG background activity is categorized as normal/sleep waves, slow/disorganized waves, asymmetric/asynchronous waves, burst-suppression waves, and attenuated/electrical silence waves, and electrographic seizure characteristics are seizure duration, clinical seizure correlate, seizure onset localization, and seizure maximal spread. Moreover, patients received carbamazepine, diazepam, phenytoin sodium, and other drug treatments combined with anti-infection treatment.

### Statistics and analyses

In the present study, continuous data were presented as median and 95% confidence intervals (CIs), and categorical data were shown as counts and percentages. The Chi-square (χ^2^) test and Fisher's exact test were used to analyze the factors associated with electrographic seizures or in-hospital outcomes. The univariate logistic regression model was established to determine the risk factors for electrographic seizures. All parameters that were significant in a univariate logistic regression model were taken into a multivariable logistic regression model in the end. The comparison of age and duration of PICU stay between the electrographic seizure group and the non-electrographic seizure group was performed by using the Mann–Whitney U test. All statistical analyses in the present study were performed using OriginPro Software version 8.5.0 (OriginLab Corporation, Northampton, MA, USA) and MedCalc Statistical Software version 15.2.2 (MedCalc Software bvba, Ostend, Belgium). A value of *p* < 0.05 was regarded as a statistically significant difference.

## Results

### Demographic characteristics of study population

**Figure 1:**
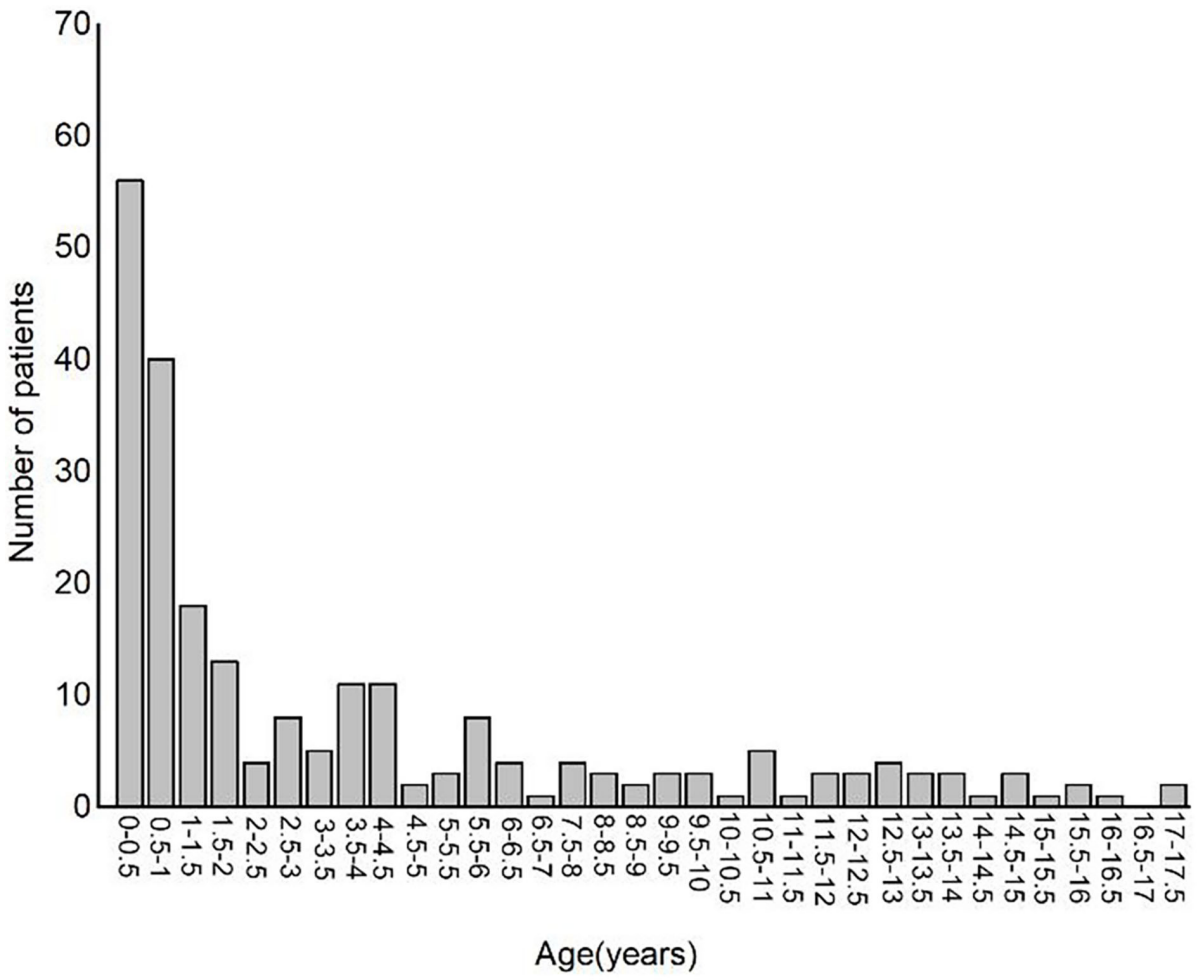
Bar plot showing patient's age distribution.

A total of 232 children were enrolled in the population, including 128 (55.17%) girls and 104 (44.83%) boys. The median duration of patient stay at the PICU was 7 days (95% CI = 6–9). The median age of the enrolled population was 1.56 ± 0.145 years. The patient age distribution is plotted in Figure 1. There were 195 (84.05%) study individuals who suffered from a neurologic disorder, 27 (11.64%) patients had CNS infection, 14 (6.03%) patients had autoimmune diseases, 28 (12.07%) patients had sepsis, 45 (19.4%) patients had structural brain impairments, 8 (3.45%) patients had previous HIE, 44 (18.97%) patients had IEM or ME, and 71 (30.6%) of patients showed prior developmental delay or intellectual disability. As many as 71.6% (166) of patients received 4 to 8 h of vEEG monitoring, 21.6% (50) of them received 8 to 16 h of vEEG monitoring, and 6.9% (15) patients received longer than 16 h of vEEG monitoring. Of the 232 patients, 26 patients had clinical seizures before being monitored for vEEG (Table 1).

**Table 1:** General and clinical factors associated with electrographic seizures.

**Variables**	**Total (*n* = 232)**	**Patients with electrographic seizures (n=88)**	**Patients without electrographic seizures (n=144)**	***p***
**Gender**				
Female *n* (%)	128 (55.17)	45 (51.14)	83 (57.64)	0.406
Male *n* (%)	104 (44.83)	43 (48.86)	61 (42.36)	
**Age(years; median)**	1.56 ± 0.145	1.47 ± 0.326	1.61 ± 0.178	0.651
**Hospitalized time in PICU(days; median)**	7.03 ± 0.472(6,9)	7 ± 0.694 (6,11)	8 ± 0.126 (6,10)	0.942
**Prior epileptic encephalopathy diagnosis**				
*n* (%)	37 (15.95)	29 (32.95)	8 (5.56)	< 0.0001
**Prior diagnosis of epilepsy diagnosis**				
*n* (%)	121 (52.16)	67 (76.14)	54 (37.5)	< 0.0001
**Clinical seizures prior to EEG**				
*n* (%)	26 (11.21)	19 (21.59)	7 (4.86)	0.0001
**Mental status at EEG beginning**				
Normal *n* (%)	10 (4.31)	2 (2.27)	8 (5.56)	0.013
Lethargic/obtunded *n* (%)	21 (9.05)	13 (14.77)	8 (5.56)	
Comatose *n* (%)	5 (2.16)	4 (4.55)	1 (0.69)	
**Neurologic disorder**				
*n* (%)	195 (84.05)	82 (93.18)	113 (78.47)	0.005
**Prior developmental delay or intellectual disability disabilityintellectual disability**				
*n* (%)	71 (30.6)	39 (44.32)	32 (22.22)	0.001
**CNS infection**				
*n* (%)	27 (11.64)	8 (9.09)	19 (13.19)	0.463
**Autoimmune diseases**				
*n* (%)	14 (6.03)	7 (7.95)	7 (4.86)	0.499
**Sepsis**				
*n* (%)	28 (12.07)	8 (9.09)	20 (13.89)	0.378
**Structural brain impairments**				
*n* (%)	45 (19.4)	13 (14.77)	32 (22.22)	0.222
**HIE**				
*n* (%)	8 (3.45)	3 (3.41)	5 (3.47)	0.73
**IEM/ME**				
*n* (%)	44 (18.97)	14 (15.91)	30 (20.83)	0.45
**Interictal epileptiform discharges**				
*n* (%)	171 (73.71)	86 (97.73)	85 (59.03)	< 0.0001
**EEG background**				
Normal/sleep *n* (%)	23 (9.91)	1 (1.14)	22 (15.28)	< 0.0001
Slow/disorganized *n* (%)	95 (40.95)	36 (40.91)	59 (40.97)	
Asymmetric/unsynchronous *n* (%)	75 (32.33)	37 (42.05)	38 (26.39)	
Burst-suppression *n* (%)	14 (6.03)	11 (12.5)	3 (2.08)	
Attenuated/electrical silence *n* (%)	21 (9.05)	1 (1.14)	20 (13.89)	

CI, confidence interval; PICU, pediatric intensive care unit; EEG, electroencephalogram; CNS, central nervous system; HIE, hypoxic ischemic encephalopathy; IEM, inborn error of metabolism; ME, mitochondrial encephalomyopathy.

### Electrographic seizure characteristics and variables associated with electrographic seizure

Table 1 shows that the variables used in the chi-square (χ^2^) test and Fisher's exact test are the significant factors associated with the occurrence of electrographic seizure (*p* < 0.05): prior epileptic encephalopathy, prior diagnosis of epilepsy, clinical seizures prior to EEG, mental status at EEG onset, neurologic disorder, prior developmental delay or intellectual disability, interictal epileptiform discharges, and EEG background. Clinical parameters that are relevant to electrographic status epilepticus in Table 2 indicated that the primary variables investigated in the study, namely, prior epileptic encephalopathy, prior epilepsy, CNS disorder, and EEG background, were not significantly associated with electrographic status epilepticus (*p* > 0.05).

**Table 2:** Analysis of related factors for status epilepticus in patients with electrographic seizures.

**Variables**	**Total (*n* = 88)**	**Patients with ES (*n* = 52)**	**Patients with status epilepticus (*n* = 36)**	***p***
**Prior epileptic encephalopathy diagnosis**				
*n* (%)	29 (32.95)	16 (30.77)	13 (36.11)	0.769
**Prior epilepsy**				
*n* (%)	67 (76.14)	38 (73.08)	29 (80.56)	0.579
**CNS disorder**				
*n* (%)	82 (93.18)	50 (96.15)	32 (88.89)	0.369
**EEG background**				
Normal/sleep *n* (%)	1 (1.14)	0	1 (2.78)	0.425
Slow/disorganized *n* (%)	36 (40.91)	20 (38.46)	16 (44.44)	
Asymmetric/unsynchronous *n* (%)	37 (42.05)	22 (42.31)	15 (41.67)	
Burst-suppression *n* (%)	11 (12.5)	8 (15.38)	3 (8.33)	
Attenuated/electrical silence *n* (%)	1 (1.14)	0	1 (2.78)	

EEG, Electroencephalogram; CNS, central never system; EEG, electroencephalogram.

The incidence of electrographic seizure in the population was 37.9% (88 of 232). Therein, 40.9% of (36 of 88) patients with electrographic seizure had electrographic status epilepticus. The characteristics of electrographic seizure are presented in Table 3. In total, 16.13, 27.42, 1.61, and 54.84% of patients were accounted for the seizure duration category of < 1 min, 1 min-5 min, 5–30 min, and >30 min, respectively. In patients with electrographic seizure, 36.36% cases were focal onset, 47.73% cases were multifocal onset, and 15.91% cases were generalized onset. In addition, 61.54% patients with electrographic seizure had bilateral seizure spread localization, 35.9% patients had unilateral seizure spread localization, and 2.56% patients had focal seizure spread localization.

**Table 3:** Electrographic seizure characteristics of 88 patients.

**Electrographic seizure characteristics**	***n* (%)**
**Seizure duration (*****n*** **=** **62)**	
< 1min	10 (16.13)
1–5 min	17 (27.42)
5–30 min	1 (1.61)
>30 min	34 (54.84)
**Clinical correlate (*****n*** **=** **44)**	
All (100%)	14 (31.82)
Most (50–99%)	12 (27.27)
Some (1–49%)	4 (9.09)
None (0%)	14 (31.82)
**Seizure onset localization (*****n*** **=** **44)**	
Focal	16 (36.36)
Multifocal	21 (47.73)
Generalized	7 (15.91)
**Seizure maximal spread localization (*****n*** **=** **39)**	
Focal	1 (2.56)
Unilateral	14 (35.9)
Bilateral	24 (61.54)

### Risk factors for electrographic seizures and in-hospital outcomes

Furthermore, we explored the risk factors for electrographic seizures in the logistic regression model, as shown in Table 4: prior epileptic encephalopathy (OR = 6.57, 95%CI = 1.91–22.59, *p* = 0.003), interictal epileptiform discharges (OR = 46.82, 95%CI = 5.31–412.86, *p* = 0.0005), slow disorganized EEG background (OR = 11.92, 95%CI = 1.31–108.71, *p* = 0.028), and burst-suppression EEG background (OR = 23.64, 95%CI = 1.71–327.57, *p* = 0.018).

**Table 4:** Risk factors for electrographic seizures.

**Variables**	**Univariate analysis**		**Multivariable analysis**	
	**OR (95% CI)**	***p***	**OR (95% CI)**	***p***
**Prior epileptic encephalopathy diagnosis**				
*n* (%)	8.36(3.6–19.36)	< 0.0001	6.57(1.91–22.59)	0.003
**Prior diagnosis of epilepsy**				
*n* (%)	5.32(2.93–9.64)	< 0.0001	1.99(0.83–4.77)	0.121
**Clinical seizures prior to EEG**				
*n* (%)	5.4(2.16–13.44)	0.0001	14.26(0.74–275.92)	0.079
**Mental status at EEG onset**				
Normal *n* (%)	Ref		Ref	
Lethargic/obtunded *n* (%)	3.09(1.22–7.8)	0.017	12.85(0.78–209.6)	0.073
Comatose *n* (%)	7.61(0.83–69.34)	0.072	-	-
**Neurologic disorder**				
*n* (%)	3.75(1.5–9.4)	0.005	1.69(0.40–7.08)	0.475
**Prior developmental delay or intellectual disability**				
*n* (%)	2.79(1.57–4.95)	0.001	1.63(0.11–22.69)	0.718
**Interictal epileptiform discharges**				
*n* (%)	56.66(7.67–418.66)	< 0.0001	46.82(5.31–412.86)	0.0005
**EEG background**				
Normal/sleep *n* (%)	Ref		Ref	
Slow/disorganized *n* (%)	13.42(1.73–103.91)	0.013	11.92(1.31–108.71)	0.028
Asymmetric/unsynchronous *n* (%)	21.42(2.75–167.15)	0.004	8.72(0.95–80.29)	0.056
Burst-suppression *n* (%)	80.67(7.5–868.23)	0.0003	23.64(1.71–327.57)	0.018
Attenuated/electrical silence *n* (%)	1.1(0.06–18.78)	0.948	-	-

OR, odds ratio; CI, confidence interval; Ref, reference; EEG, electroencephalogram.

In the present study, 77.16% (179 of 232) of patients had improved condition and were discharged from the hospital, 14.66% (34 of 232) of patients had not improved and were voluntarily discharged from the hospital, and 7.76% (18 of 232) of patients died. The associated factors for in-hospital outcomes are presented in Table 5. It was found that clinical variables, such as gender, age, prior epileptic encephalopathy, prior diagnosis of epilepsy, seizure category, neurologic disorder, CNS infection, structural brain impairments, HIE, and interictal epileptiform discharges, were not related to in-hospital outcomes (*p* > 0.05). Moreover, patients with sepsis and/or abnormal EEG background in PICU were more likely to face an unfavorable in-hospital outcome (*p* < 0.05).

**Table 5:** Association between clinical variables and in-hospital outcomes.

**Variables**	**Total (*n* = 231)**	**Good outcome (*n* =1 79)^a^**	**Unfavorable outcome (*n* = 52)^b^**	***p***
**Gender**				
Female *n* (%)	128 (55.41)	100 (55.87)	28 (53.85)	0.921
Male *n* (%)	101 (44.59)	79 (44.23)	24 (46.15)	
**Age (years; median, 95% CI)**	1.56 (1.12, 2.44)	1.47 (0.96, 2.96)	1.61 (1.06, 2.68)	0.823
**Prior epileptic encephalopathy diagnosis**				
*n* (%)	37 (16.02)	32 (17.88)	5 (9.62)	0.224
**Prior diagnosis of epilepsy**				
*n* (%)	121 (52.38)	98 (57.75)	23 (44.23)	0.238
**Seizure category**				
No *n* (%)	144 (62.34)	108 (60.34)	36 (69.23)	0.497
Seizures *n* (%)	51 (22.08)	42 (23.46)	9 (17.31)	
Status epilepticus *n* (%)	36 (15.58)	29 (16.2)	7 (13.46)	
**Clinical seizures prior to EEG**				
*n* (%)	13 (5.63)	11 (6.15)	2 (3.85)	0.729
**Neurologic disorder**				
*n* (%)	194 (83.98)	153 (85.47)	41 (78.85)	0.351
**Prior developmental delay or intellectual disability**				
*n* (%)	71 (30.74)	56 (31.28)	15 (28.85)	0.419
**CNS infection**				
*n* (%)	27 (11.69)	21 (11.73)	6 (11.54)	0.836
**Autoimmune diseases**				
*n* (%)	14 (6.06)	13 (7.26)	1 (1.92)	0.276
**Sepsis**				
*n* %)	28 (12.12)	17 (9.5)	11 (21.15)	0.043
**Structural brain impairments**				
*n* (%)	45 (19.48)	34 (18.99)	11 (21.15)	0.883
**HIE**				
*n* (%)	7 (3.03)	4 (2.23)	3 (5.77)	0.396
**IEM/ME**				
*n* (%)	44 (19.05)	29 (16.2)	15 (28.85)	0.065
**Interictal epileptiform discharges**				
*n* (%)	170 (73.59)	135 (75.42)	35 (67.31)	0.323
**EEG background**				
Normal/sleep *n* (%)	23 (9.96)	22 (12.29)	1 (1.92)	0.0005
Slow/disorganized *n* (%)	95 (41.13)	79 (44.13)	16 (30.77)	0.0006
Asymmetric/unsynchronous *n* (%)	75 (32.47)	60 (33.52)	15 (28.85)	0.0016
Burst-suppression *n* (%)	13 (5.63)	9 (5.03)	4 (7.69)	0.0085
Attenuated/electrical silence *n* (%)	21 (9.09)	6 (3.35)	15 (28.85)	0.0008

^a^Improved condition and discharged; ^b^Not improved condition or dead. EEG, electroencephalogram; CNS, central nervous system; HIE, hypoxic ischemic encephalopathy; IEM, inborn error of metabolism; ME, mitochondrial encephalomyopathy.

## Discussion

The present study retrospectively analyzed the occurrence of electrographic seizures and EEG background among 232 children who underwent bedside vEEG monitoring in PICU of a tertiary hospital in China. The results indicate that the incidence of electrographic seizures was 37.9%, of which 40.9% electrographic seizures were status epilepticus. In addition, 88.4% patients had an abnormal initial EEG background.

The categories of admission diagnoses in PICU and the length of ICU stay are quite different from those in an adult or neonatal intensive care unit (ICU). Acute respiratory and cardiac diseases, trauma, and seizures are the most common admission diagnoses in PICU (16, 17). However, in this study, a large proportion of patients had varying degrees of primary neurologic disorder, such as ME, previous HIE, other structural brain impairments, CNS infection, and IEM, with the reasoning put forth that patients with these diseases have a poor mental state and were thus considered eligible for the study. In the present study, the proportion of electrographic seizures in only 31.8% of patients was completely correlated with clinical signs, which illustrated the significance of vEEG monitoring in PICU for the detection of subclinical electrographic seizures. Electrographic seizures without clear clinical signs were regarded as subclinical electrographic seizures in our study. Moreover, a reported research indicated that 75% of critically ill children with electrographic seizures who underwent CEEG have complete non-convulsive seizures (NCS) (2). Another study on seizures in infants also showed that only 21% of seizures detected by EEG are related to clinical signs (15). All these findings revealed that the majority of seizures in critically ill patients are subclinical seizures without obvious clinical signs, which can only be determined by EEG monitoring. Moreover, the recruitment of patients in our study was sequential and had no bias for any specific diagnosis.

The confirmation of risk factors for electrographic seizures can be potential indicators to evaluate the condition of children in PICU and draw different monitoring strategies (18). However, distinguishing patients with a high risk of seizure is of benefit for the best use of limited EEG resources. Prior epileptic encephalopathy, presence of interictal epileptiform discharges, and abnormal initial EEG background are the risk factors for electrographic seizures determined by this study. Clinical and electrographic risk factors for seizures, including clinical seizures, brain impairment induced by multiple reasons, epileptiform discharges, and abnormal EEG background activity, have been identified by several studies (2, 5, 19, 20). Although young age has been regarded as one of the risk factors for seizures in partial studies (5, 7, 8), it is not a significant parameter in some other literature (2, 19, 20). Thus, a study with an age-balanced population can be helpful to illustrate the association between age and electrographic seizures.

Although there is no common opinion regarding the association between electrographic seizures and clinical outcomes, some researchers are striving to prove it. A study on 204 comatose children concluded that seizures are associated with an unfavorable outcome in survivor patients, but not in dead cases (11). Other studies also reported that status epilepticus, but not all epileptic seizure types, is related to higher in-hospital deaths (4, 7, 8, 12, 21, 22). There may be a connection between seizure burden and poor outcomes, but early seizure detection by EEG and effective antiseizure treatment may improve the condition reversely (23). In the present study, sepsis and abnormal EEG background activity are the associated factors for in-hospital unfavorable outcomes such as unimproved condition and death. There is no significant association between seizure presence and unfavorable in-hospital outcomes in current research.

There are several limitations to this study. First, the size of the studied population is insufficient for a comprehensive in-hospital outcome analysis. It is very limited for determining the connection of mortality and electrographic seizures. Second, the population was enrolled in a single institution. Since a large proportion of the study individuals (84.05%) in the PICU had neurologic disorders, an admission-rate bias was present for this research. Third, the limited total durations of vEEG monitoring might not detect electrographic seizures completely. The clinical application of vEEG should consider both the misdiagnosis of abnormal EEG activities and the reasonable use of healthcare medical resource. Fourth, it is possible that substantial bias was introduced into this study, because it is unlikely to monitor patients without neurological symptoms or risk for neurological complications. Future research is necessary to investigate how the early detection of electrographic seizures by vEEG affects the clinical outcomes in critically ill patients.

In conclusion, this study indicates that the occurrence of electrographic seizures is over one-third of the population in PICU. vEEG is an efficient method to determine electrographic seizures in children. Abnormal EEG background activities are indicators both for the occurrence of electrographic seizures and for unimproved condition or even in-hospital deaths.

## Data availability statement

The original contributions presented in the study are included in the article/supplementary material, further inquiries can be directed to the corresponding author/s.

## Ethics statement

The studies involving human participants were reviewed and approved by the Ethics Committee of Peking University First Hospital (2021KY422). Written informed consent to participate in this retrospective study was not considered necessary by the Ethics Committee.

## Author contributions

YWu and YWa were the guarantors of integrity of the entire study and undertook study design. YWu gave the definition of intellectual content and undertook manuscript review. QG and ZY gave data acquisition. TS undertook data analysis, statistical analysis, and manuscript preparation and editing. All authors contributed to the article and approved the submitted version.

## Conflict of interest

The authors declare that the research was conducted in the absence of any commercial or financial relationships that could be construed as a potential conflict of interest.

## Publisher's note

All claims expressed in this article are solely those of the authors and do not necessarily represent those of their affiliated organizations, or those of the publisher, the editors and the reviewers. Any product that may be evaluated in this article, or claim that may be made by its manufacturer, is not guaranteed or endorsed by the publisher.

## References

[1] Towne AR Waterhouse EJ Boggs JG Garnett LK Brown AJ Smith JR. Prevalence of nonconvulsive status epilepticus in comatose patients. Neurology. (2000) 54:340–5. 10.1212/WNL.54.2.340 10668693

[2] Jette N Claassen J Emerson RG Hirsch LJ. Frequency and predictors of nonconvulsive seizures during continuous electroencephalographic monitoring in critically ill children. Arch Neurol. (2006) 63:1750–5. 10.1001/archneur.63.12.1750 17172615

[3] Shahwan A Bailey C Shekerdemian L Harvey AS. The prevalence of seizures in comatose children in the pediatric intensive care unit: a prospective video-EEG study. Epilepsia. (2010) 51:1198–204. 10.1111/j.1528-1167.2009.02517.x 20163439

[4] Abend NS Gutierrez-Colina AM Topjian AA Zhao H Guo R Donnelly M. Nonconvulsive seizures are common in critically ill children. Neurology. (2011) 76:1071–7. 10.1212/WNL.0b013e318211c19e 21307352 PMC3068008

[5] Williams K Jarrar R Buchhalter J. Continuous video-EEG monitoring in pediatric intensive care units. Epilepsia. (2011) 52:1130–6. 10.1111/j.1528-1167.2011.03070.x 21671924

[6] Privitera M Hoffman M Moore JL Jester D. EEG detection of nontonic-clonic status epilepticus in patients with altered consciousness. Epilepsy Res. (1994) 18:155–66. 10.1016/0920-1211(94)90008-6 7957038

[7] Abend NS Arndt DH Carpenter JL Chapman KE Cornett KM Gallentine WB. Electrographic seizures in pediatric ICU patients: cohort study of risk factors and mortality. Neurology. (2013) 81:383–91. 10.1212/WNL.0b013e31829c5cfe 23794680 PMC3772834

[8] Abend NS Chapman KE Gallentine W Goldstein J Hyslop AE Loddenkemper T. Electroencephalographic monitoring in the pediatric intensive care unit. Curr Neurol Neurosci Reports. (2013) 13:330. 10.1007/s11910-012-0330-3 23335026 PMC3569710

[9] Bozarth XL McGuire J Novotny E. Current Status of continuous electroencephalographic monitoring in critically ill children. Pediatr Neurol. (2019) 101:11–7. 10.1016/j.pediatrneurol.2019.07.012 31493974

[10] Alehan FK Morton LD Pellock JM. Utility of electroencephalography in the pediatric emergency department. J Child Neurol. (2001) 16:484–7. 10.1177/088307380101600704 11453443

[11] Kirkham FJ Wade AM McElduff F Boyd SG Tasker RC Edwards M. Seizures in 204 comatose children: incidence and outcome. Intensive Care Med. (2012) 38:853–62. 10.1007/s00134-012-2529-9 22491938 PMC3338329

[12] Topjian AA Gutierrez-Colina AM Sanchez SM Berg RA Friess SH Dlugos DJ. Electrographic status epilepticus is associated with mortality and worse short-term outcome in critically ill children. Crit Care Med. (2013) 41:215–23. 10.1097/CCM.0b013e3182668035 23164815 PMC3531581

[13] Sanchez SM Carpenter J Chapman KE Dlugos DJ Gallentine WB Giza CC. Pediatric ICU EEG monitoring: current resources and practice in the United States and Canada. J Clin Neurophysiol Am Electroencephal Soc. (2013) 30:156–60. 10.1097/WNP.0b013e31827eda27 23545766 PMC3616267

[14] Young GB Jordan KG Doig GS. An assessment of nonconvulsive seizures in the intensive care unit using continuous EEG monitoring: an investigation of variables associated with mortality. Neurology. (1996) 47:83–9. 10.1212/WNL.47.1.83 8710130

[15] Clancy RR Legido A Lewis D. Occult neonatal seizures. Epilepsia. (1988) 29:256–61. 10.1111/j.1528-1157.1988.tb03715.x 3371282

[16] Ruttimann UE Patel KM Pollack MM. Length of stay and efficiency in pediatric intensive care units. J Pediatr. (1998) 133:79–85. 10.1016/S0022-3476(98)70182-9 9672515

[17] Khilnani P Sarma D Singh R Uttam R Rajdev S Makkar A. Demographic profile and outcome analysis of a tertiary level pediatric intensive care unit. Indian J Pediat. (2004) 71:587–91. 10.1007/BF02724117 15280607 PMC7102310

[18] Gutierrez-Colina AM Topjian AA Dlugos DJ Abend NS. Electroencephalogram monitoring in critically ill children: indications and strategies. Pediatr Neurol. (2012) 46:158–61. 10.1016/j.pediatrneurol.2011.12.009 22353290 PMC3286021

[19] McCoy B Sharma R Ochi A Go C Otsubo H Hutchison JS. Predictors of non-convulsive seizures among critically ill children. Epilepsia. (2011) 52:1973–8. 10.1111/j.1528-1167.2011.03291.x 22003955

[20] Greiner HM Holland K Leach JL Horn PS Hershey AD Rose DF. Nonconvulsive status epilepticus: the encephalopathic pediatric patient. Pediatrics. (2012) 129:e748–55. 10.1542/peds.2011-2067 22331332 PMC9923578

[21] Lambrechtsen FA Buchhalter JR. Aborted and refractory status epilepticus in children: a comparative analysis. Epilepsia. (2008) 49:615–25. 10.1111/j.1528-1167.2007.01465.x 18093148

[22] Abend NS. Electrographic status epilepticus in children with critical illness: Epidemiology and outcome. Epilepsy and behavior: EandB. (2015) 49:223–7. 10.1016/j.yebeh.2015.03.007 25944114 PMC4536159

[23] Pinchefsky EF Hahn CD. Outcomes following electrographic seizures and electrographic status epilepticus in the pediatric and neonatal ICUs. Curr Opin Neurol. (2017) 30:156–64. 10.1097/WCO.0000000000000425 28118303

